# Survival Significance of Number of Positive Lymph Nodes in Oral Squamous Cell Carcinoma Stratified by p16

**DOI:** 10.3389/fonc.2021.545433

**Published:** 2021-03-04

**Authors:** Peng Li, Qigen Fang, Yanjie Yang, Defeng Chen, Wei Du, Fei Liu, Ruihua Luo

**Affiliations:** ^1^Department of Head Neck and Thyroid, Affiliated Cancer Hospital of Zhengzhou University, Henan Cancer Hospital, Zhengzhou, China; ^2^Department of Oral Medicine, The First Affiliated Hospital of Zhengzhou University, Zhengzhou, China

**Keywords:** oral squamous cell carcinoma, AJCC classification, number of positive lymph nodes, survival analysis, p16

## Abstract

**Objectives:** To analyze the significance of the number of positive lymph nodes in oral squamous cell carcinoma (SCC) stratified by p16.

**Methods:** A total of 674 patients were retrospectively enrolled and divided into 4 groups based on their number of positive lymph nodes (0 vs. 1–2 vs. 3–4 vs. ≥5). The Kaplan-Meier method was used to calculate the disease-free survival (DFS) and disease-specific survival (DSS) rates. Cox model was used to evaluate the independent risk factor.

**Results:** p16 showed positivity in 85 patients with a rate of 12.6%. In patients with p16 negativity, the 5-year DFS rates were 52%, 39%, and 21% in patients with 0, 1–2, and 3–4 positive lymph nodes, respectively, in patients with ≥5 positive lymph nodes, all patients developed recurrence within 2 years after operation, the difference was significant; the 5-year DSS rates were 60, 38, and 18% in patients with 0, 1–2, and 3–4 positive lymph nodes, respectively, in patients with ≥5 positive lymph nodes, all patients died within 4-years after operation. The difference was significant. In p16 positivity patients, the 3-year DFS rates were 41% and 17% in patients with 0–2 and ≥3 positive lymph nodes, respectively, the difference was significant; the 3-year DSS rates were 84 and 46% in patients with 0–2 and ≥3 positive lymph nodes, the difference was significant.

**Conclusions:** The number of positive lymph nodes is significantly associated with the survival in oral SCC, its survival effect is not affected by p16 status.

## Introduction

Oral squamous cell carcinoma (SCC) is the most common malignancy in the head and neck, and the mainstay of treatment is curative surgery followed by adjuvant treatment (1). Although there has been great progress in medical science, the prognosis of oral SCC has not apparently improved with a 5-year overall survival rate of about 40% (2–4). The most important prognostic factor is cervical nodal metastasis, the survival would decrease by half even if there is only one positive lymph node (5). Much effort has been made to formulate a reliable neck lymph node classification for better guiding treatment and predicting prognosis. The newest version of AJCC classification takes the size, number, extracapsular spread (ECS), and laterality of positive nodes into consideration during the cervical nodal status definition (6). However, a number of researchers have noted that this classification fails to detect the survival difference between N1 and N2a disease (7), and also between N2b and N2c disease (8). Thus, a proposed nodal system based on the number of positive lymph nodes is suggested, and it is verified to be superior to the 8th AJCC classification (9–11).

HPV-induced cancer is attracting more and more attention, and it contributes to at least 70% of the newly diagnosed oropharynx SCC. p16 over-expression is significantly associated with HPV infection (12), and it usually carries a favorable prognosis in oropharynx SCC. However, the reported rates of HPV infection and p16 over-expression as well as its impact on prognosis in oral SCC varies greatly (13). Therefore, in the current study, we aimed to analyze the significance of the number of positive lymph nodes in oral SCC stratified by p16.

## Patients and Methods

### Ethical Consideration

Henan Cancer Hospital institutional research committee approved our study, and all participants signed an informed consent agreement. All methods were performed in accordance with the relevant guidelines and regulations. All procedures performed in studies involving human participants were in accordance with the ethical standards of the institutional and/or national research committee and with the 1964 Helsinki declaration and its later amendments or comparable ethical standards.

### Patient Selection

Medical records of patients undergoing surgical treatment for primary oral SCC between January 2013 and December 2019 were retrospectively enrolled, included patients needed to meet the following criteria: there was no history of other cancer; there was enough tissue available for HPV analysis; the patient received neck dissection; the number of lymph nodes examined was not <10. Demography and pathologic information, and TNM stage based on the 8th AJCC classification as well as follow-up data was extracted and analyzed.

### Important Variable Definition

Drinkers were defined as those who consumed at least one alcoholic drink per day for at least 1 year, and smokers were defined as those who smoked on a daily basis or had quit smoking for <5 years (3), perineural invasion (PNI) was considered to be present if tumor cells were identified within the perineural space and/or nerve bundle; lymphovascular infiltration was positive if tumor cells were noted within the lymphovascular channels (14). The pathologic depth of invasion (DOI) was measured from the level of the adjacent normal mucosa to the deepest point of tumor infiltration, regardless of the presence or absence of ulceration (6). Extracapsular spread (ECS) was positive if there were tumor cells out of the capsular of the positive lymph node (15).

### Immunohistochemical (IHC) Analysis

From July 2013, routine immunohistochemical analysis of p16 was performed for every oral SCC patient. Level of positivity of p16 over expression was consistent with previous studies well (16): 0-+, defined as <25% tumor staining; ++, defined as 25–50% tumor staining; +++, defined as 50–75% tumor staining; and ++++: defined as more than 75% tumor staining. Tumors with level +++ and ++++ classified as positive p16.

### HPV Assessment

From July 2013, HPV detection was selectively performed for oral SCC patients in our cancer center by fresh tumor tissue. DNA was extracted using TIANcombi DNA Lyse&Det PCR Kit (TIANGEN Cooperation, Beijing, China), and then submitted to real-time PCR with the INNO-LIPA HPV Genotyping Extra System® kit (Innogenetics), it could detect 7 low-risk HPV types (6, 11, 40, 43, 44, 54, 70), 3 indeterminate-risk types (69, 71, 74), and 18 high risk HPV types (16, 18, 26, 31, 33, 35, 39, 45, 51, 52, 53, 56, 58, 59, 66, 68, 73, 82). For paraffin-embedded tissue, at least five 10-um thick slices were used for DNA extraction by TIANcombi DNA Lyse&Det PCR Kit (TIANGEN Cooperation, Beijing, China) according to the instruction, the following procedures were similar with above-mentioned description.

### Treatment Proposal

In our cancer center, preoperative systemic examinations of ultrasound, CT/MRI and/or PET-CT was performed for every patient. Complete resection of primary tumor was achieved with at least 1 cm margin, a free flap or pedicled flap was used to close the defect if necessary. For a cN0 neck, a dissection of level 1 to 3/4 was performed, for a cN+ neck, a modified radical or radical neck dissection of level 1 to 5 was performed. Adjuvant treatment was suggested if there was presence of T3/4 disease, pathologic cervical disease, PNI, LVI, positive margin, and ECS. After discharging, the patient was followed every 3 months for the first 2 years, every 6 months for the third to fifth year, and then once per year. If there was suspicion of disease recurrence, active inference was taken.

### Statistic Analysis

The cut-off value of positive lymph nodes was defined according to previous studies (8, 10, 17), the patients were divided into four groups based on the four knots (0 vs. 1–2 vs. 3–4 vs. ≥5). The difference among the four groups was compared using the Chi-square test. However, owing to the small sample size of patients with p16 positivity, these patients were divided into two groups (0–2 vs. ≥3), and also because of their limited follow-up time, prognostic difference of the two groups was compared using the 3-year survival rate. The study endpoints were disease-free survival (DFS) and the disease specific survival (DSS), and they were calculated by the Kaplan-Meier method. The survival time of DFS was calculated from the date of surgery to the date of first locoregional recurrence or distant metastasis or the last follow-up. The survival time of DSS was calculated from the date of surgery to the date of cancer-caused death or the last follow-up. The factors which were significant in univariate analysis were then analyzed in the Cox proportional hazards model to find out the independent factor. The Harrell's C-concordance index was used to compare the model fitness between number of positive lymph nodes model and the 8th AJCC classification, where the higher the value, the better the discrimination among subgroups (18). All statistical analyses were performed using SPSS 20.0, a value of *p* < 0.05 was considered to be significant.

## Results

### Demography and Pathologic Data

A total of 674 patients were enrolled for analysis, there were 517 (76.7%) male and 157 (23.3%) female, the mean age was 57.5 years with a range from 32 years to 78 years. Smoker and drinker were noted in 519 (77.0%) patients and 387 (57.4%) patients, respectively.

Primary sites were characterized as tongue in 248 (36.8%) patients, buccal in 177 (26.3%) patients, gingiva in 132 (19.6%) patients, and the floor of the mouth in 117 (17.4%) patients. Pathologic tumor stages were distributed as T1 in 118 (17.5%) patients, T2 in 267 (39.6%) patients, T3 in 189 (28.0%) patients, and T4 in 100 (14.8%) patients. The mean pathologic DOI was 9.8 mm with a range from 1.4 to 24.5 mm. Tumor differentiation was distributed as well in 266 (39.5%) patients, moderate in 300 (44.5%) patients, and poor in 108 (16.0%) patients. PNI and LVI was presented in 273 (40.5%) patients and 234 (34.7%) patients, respectively. Positive margin occurred in 35 (5.2%) patients.

The mean number of lymph nodes examined was 21.5 with a range from 12 to 47. Positive cervical disease occurred in 289 (42.9%) patients, and pathologic neck lymph node stages were distributed as N0 in 385 (57.1%) patients, N1 in 103 (15.3%) patients, N2 in 107 (15.9%) patients, and N3 in 79 (11.7%) patients. In the 289 patients with cervical nodal metastasis, 130 (45.0%) patients had one positive lymph node, 50 (17.3%) patients had two positive lymph nodes, 40 (13.8%) patients had three positive lymph nodes, 32 (11.1%) patients had four positive lymph nodes, 20 (6.9%) patients had five positive lymph nodes, and 17 (5.9%) patients had more than 5 positive lymph nodes. ECS occurred in 79 (11.7%) patients (Table 1).

**Table 1:** Demography and pathologic data in the 674 patients with oral squamous cell carcinoma.

**Variables**	***N* (%)**
**Age**	
<40	78 (11.6%)
≥40	596 (88.4%)
**Gender**	
Male	517 (76.7%)
Female	157 (23.3%)
Smoker	519 (77.0%)
Drinker	387 (57.4%)
**Primary site**	
Tongue	248 (36.8%)
Buccal	177 (26.3%)
Gingiva	132 (19.6%)
The floor of the mouth	117 (17.4%)
**Pathologic tumor stage**	
T1+T2	385 (57.1%)
T3+T3	289 (42.9%)
**Tumor differentiation**	
Well	266 (39.5%)
Moderate	300 (44.5%)
Poor	108 (16.0%)
Perineural invasion	273 (40.5%)
Lymphovascular invasion	234 (34.7%)
Positive margin	35 (5.2%)
**Pathologic neck lymph node stage**	
N0	385 (57.1%)
N1	103 (15.3%)
N2	107 (15.9%)
N3	79 (11.7%)

### HPV and p16 Test

HPV show positivity in 69 patients with a rate of 10.2%, in whom 30 (43.5%) patients had a tumor arising from the tongue, 15 (21.7%) cases from the buccal, 10 (14.5%) cases from the gingiva, and 14 (20.2%) cases from the floor of the mouth. 7 (10.1%) of the 69 patients also showed p16 positivity.

p16 showed positivity in 85 patients with a rate of 12.6%, in whom 50 (61.0%) patients had a tumor arising from the tongue, 8 (9.4%) cases from the buccal, 7 (8.2%) cases from the gingiva, and 20 (23.5%) cases from the floor of the mouth. 8 (9.4%) of the 85 patients also showed HPV positivity.

### Comparison Among the Four Groups

The four groups had similar distribution regarding age (*p* = 0.235), drinker status (*p* = 0.893), and HPV positivity (*p* = 0.946). There was significant difference of distribution of gender (*p* = 0.013), smoker status (*p* < 0.001), primary site (*p* < 0.001), pathologic tumor stage (*p* < 0.001), tumor differentiation (*p* < 0.001), PNI (*p* < 0.001), LVI (*p* < 0.001), ECS (*p* < 0.001), positive margin (*p* < 0.001), neck lymph node stage (*p* < 0.001), and p16 positivity(*p* = 0.024) (Table 2). Patients with greater number of positive lymph nodes tended to be a smoking man with SCC arising from the tongue or the floor of the mouth. Adverse pathologic characteristics including high tumor stage, presence of PNI, LVI, and ECS, and cervical nodal disease were more frequent in patients having more than 5 positive lymph nodes. Additionally, p16 positivity was associated with greater number of positive lymph nodes.

**Table 2:** Comparison of clinical and pathologic variables among patients with different numbers of positive lymph nodes.

**Variables**	**Number of positive lymph nodes**				***p***
	**0 (*n* = 385)**	**1–2 (*n* = 180)**	**3–4 (*n* = 72)**	**≥5 (*n* = 37)**	
**Age**					
<40	38 (9.9%)	22 (12.2%)	13 (18.1%)	5 (13.5%)	
≥40	347 (90.1%)	158 (87.8%)	59 (81.9%)	32 (86.5%)	0.235
**Sex**					
Male	279 (72.5%)	144 (80.0%)	61 (84.7%)	33 (89.2%)	
Female	106 (27.5%)	36 (20.0%)	11 (15.3%)	4 (10.8%)	0.013
Smoker	277 (71.9%)	143 (79.4%)	62 (86.1%)	37 (100%)	<0.001
Drinker	225 (58.4%)	100 (55.6%)	42 (58.3%)	20 (54.1%)	0.893
**Primary site**					
Tongue	114 (29.6%)	76 (42.2%)	35(48.6%)	23 (62.2%)	
Buccal	122 (31.7%)	42 (23.3%)	11 (15.3%)	2 (5.4%)	
Gingiva	90 (23.4%)	30 (16.7%)	10 (13.9%)	2 (5.4%)	
The floor of the mouth	59 (15.3%)	32 (17.8%)	16 (22.2%)	10 (27.0%)	<0.001
**Pathologic tumor stage**					
T1+T2	253 (65.7%)	102 (56.7%)	25 (34.7%)	5 (13.5%)	
T3+T4	132 (34.3%)	78 (43.3%)	47 (65.3%)	32 (86.5%)	<0.001
**Tumor differentiation**					
Well	197 (51.2%)	50 (27.8%)	15 (20.8%)	4 (10.8%)	
Moderate + poor	188 (48.8%)	130 (72.2%)	57 (79.2%)	33 (89.2%)	<0.001
Perineural invasion	116 (30.1%)	85 (47.2%)	42 (58.3%)	30 (81.1%)	<0.001
Lymphovascular invasion	101 (26.2%)	61 (33.9%)	45 (62.5%)	27 (73.0%)	<0.001
Positive margin	8 (2.1%)	13 (7.2%)	8 (11.1%)	6 (16.2%)	<0.001
Extracapsular spread	–	26 (14.4%)	26 (36.1%)	27 (73.0%)	<0.001
**Neck lymph node stage**					
N1	–	103 (57.2%)	0	0	
N2	–	59 (32.8%)	36 (50.0%)	12 (32.4%)	
N3	–	18 (10.0%)	36 (50.0%)	25 (67.6%)	<0.001
HPV positivity	37 (9.6%)	20 (11.1%)	8 (11.1%)	4 (10.8%)	0.946
p16 positivity	41 (10.6%)	22 (12.2%)	12 (16.7%)	10 (27.0%)	0.024

During our follow-up, with a mean time of 40.0 months, a total of 463 patients received adjuvant treatment, of which 286 patients received radiotherapy, 177 patients received chemoradiotherapy. Recurrence occurred in 340 patients: 252 patients had locoregional recurrence, and 88 patients had concurrent locoregional recurrence and distant metastasis. Hundred patients received salvage surgical treatment, and the rest received palliative chemotherapy. Two hundred and sixty seven patients died of the disease. The overall 5-year DFS and DSS rates were 41 and 41%, respectively.

**Figure 1:**
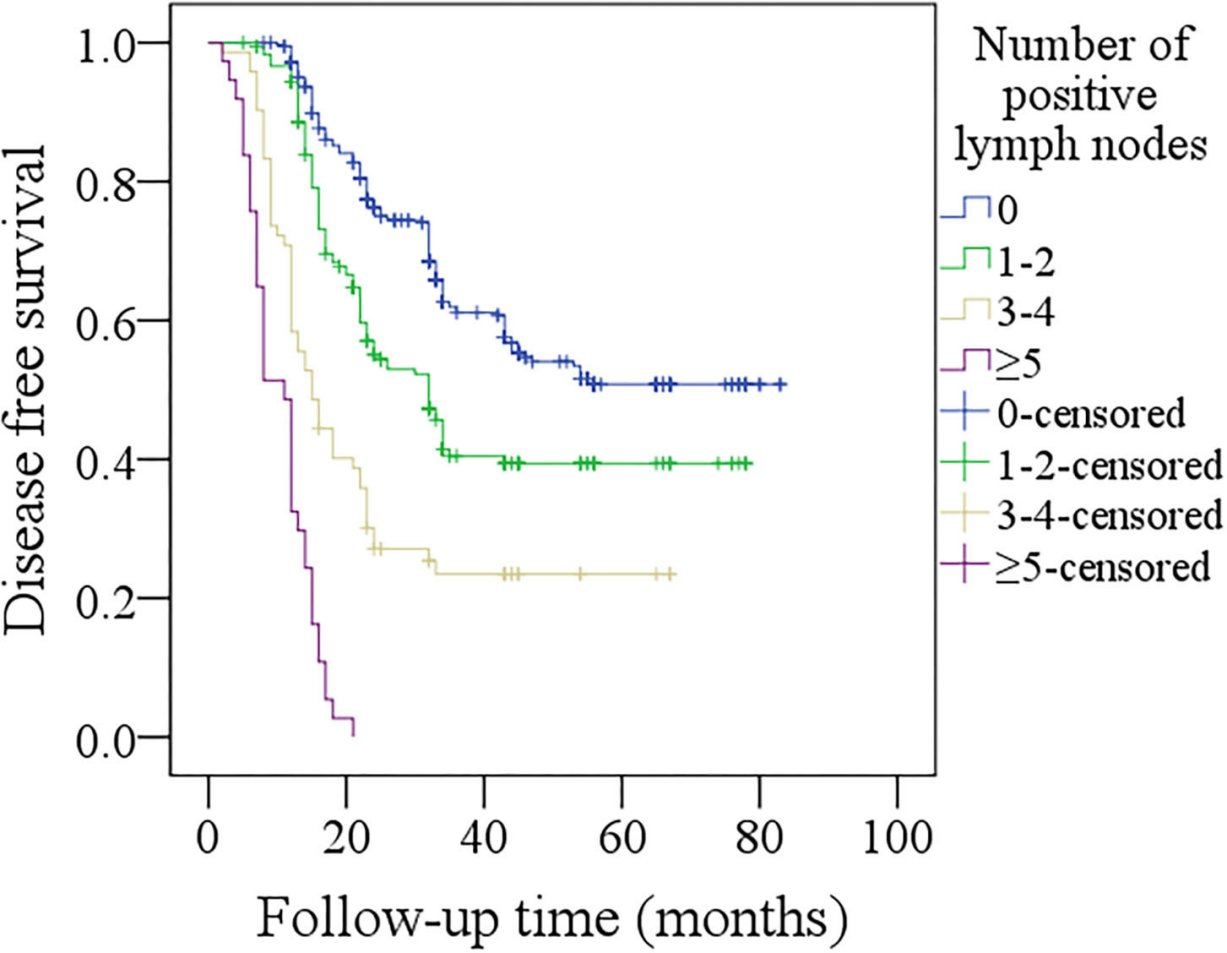
Comparison of disease-free survival among patients with different numbers of positive lymph nodes (*p* < 0.001).

In patients with no positive lymph nodes, the 5-year DFS rate was 49%, in patients with 1–2 positive lymph nodes, the 5-year DFS rate was 39%, in patients with 3–4 positive lymph nodes, the 5-year DFS rate was 23%, in patients with ≥5 positive lymph nodes, all patients developed recurrence within 2 years after operation. The difference was significant (Figure 1, *p* < 0.001). In further Cox model analysis, the factors of smoker, the number of positive lymph nodes, primary site, pathologic tumor stage, tumor differentiation, PNI, LVI, neck lymph node stage, and p16 were significantly associated with the DFS (Table 3). The Harrell's C-concordance index for number of positive lymph nodes system and the 8th AJCC neck lymph node classification was 0.7312 and 0.7299.

**Table 3:** Univariate and Cox model analyses of the disease-free survival in the 674 patients.

**Variables**	**Univariate**	**Cox model**	
	***p***	***p***	**HR [95% CI]**
Age (<40 vs. ≥40)	0.156		
Sex (Male vs. female)	0.342		
Smoker	<0.001	<0.001	1.461 [1.197–1.998]
Drinker	0.471		
**Primary site**			
Tongue + The mouth floor vs. others	<0.001	<0.001	2.476 [1.227–3.471]
**Pathologic tumor stage**			
T3+T4 vs. T1+T2	<0.001	<0.001	3.446 [1.385–6.331]
**Tumor differentiation**			
Moderate + poor vs. well	<0.001	<0.001	1.998 [1.264–3.558]
Perineural invasion	<0.001	<0.001	2.363 [1.277–4.338]
Lymphovascular invasion	<0.001	<0.001	2.255 [1.304–4.264]
Neck lymph node stage	<0.001		
N0			
N1		<0.001	1.685 [1.125–2.138]
N2		<0.001	2.453 [1.773–3.467]
N3		<0.001	3.007 [2.162–6.487]
Number of lymph node examined			
<22 vs. ≥22	0.267		
HPV positivity	0.993		
p16 positivity	<0.001	<0.001	1.565 [1.183–2.021]
Positive margin	<0.001	<0.001	1.996 [1.317–2.778]
Number of positive lymph nodes	<0.001		
0			
1–2		<0.001	1.981 [1.241–2.525]
3–4		<0.001	3.126 [2.612–4.178]
≥5		<0.001	5.453 [4.431–8.465]

**Figure 2:**
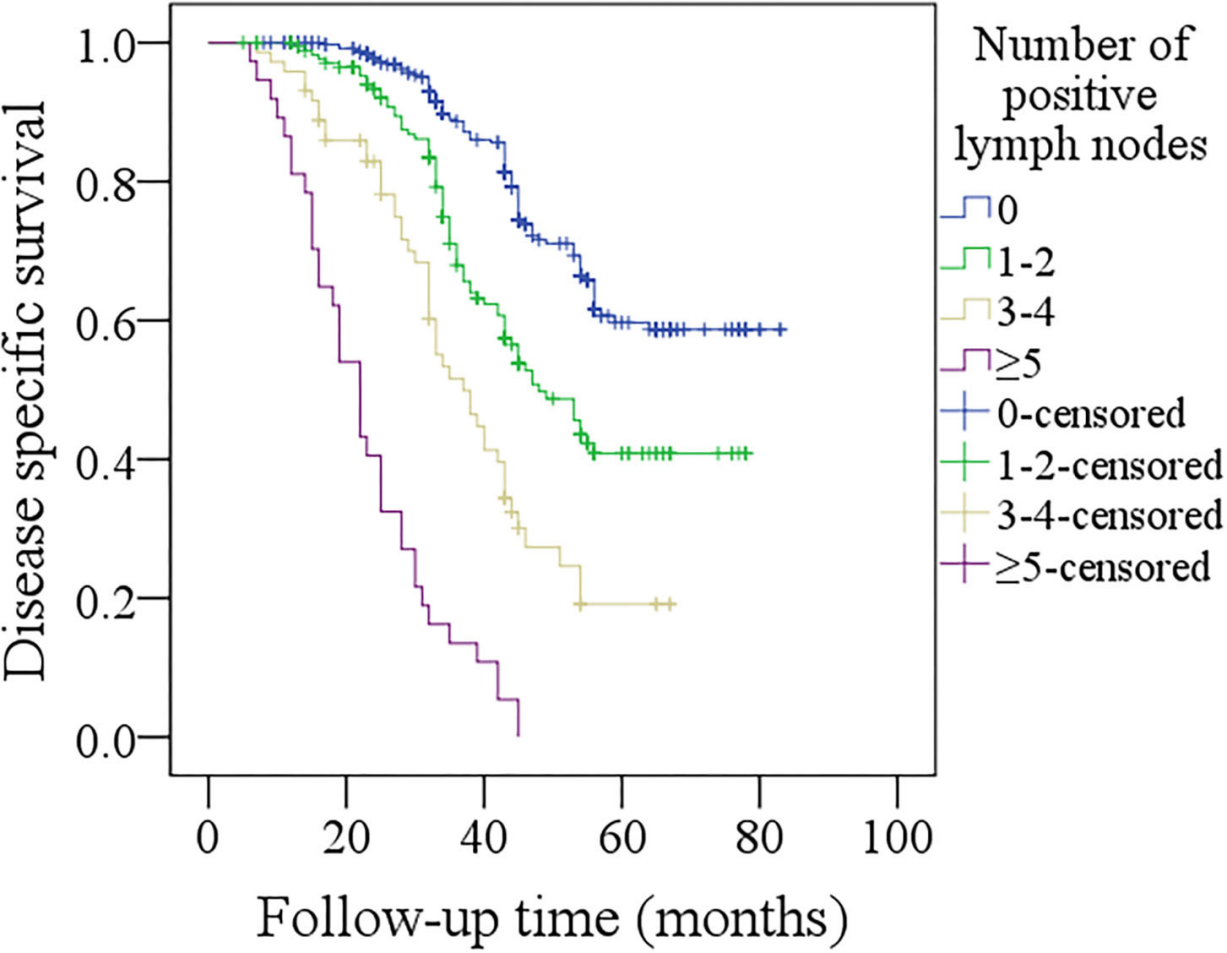
Comparison of disease-specific survival among patients with different numbers of positive lymph nodes (*p* < 0.001).

In patients with no positive lymph nodes, the 5-year DSS rate was 57%, in patients with 1–2 positive lymph nodes, the 5-year DSS rate was 39%, in patients with 3–4 positive lymph nodes, the 5-year DSS rate was 17%, in patients with ≥5 positive lymph nodes, all patients died of the disease within 4 years after operation. The difference was significant (Figure 2, *p* < 0.001). In further Cox model analysis, the factors of smoker status, the number of positive lymph nodes, primary site, pathologic tumor stage, tumor differentiation, PNI, LVI, neck lymph node stage, and p16 were significantly associated with the DSS (Table 4). The Harrell's C-concordance index for number of positive lymph nodes system and the 8th AJCC neck lymph node classification was 0.7200 and 0.7186.

**Table 4:** Univariate and Cox model analyses of the disease-specific survival in the 674 patients.

**Variables**	**Univariate**	**Cox model**	
	***p***	***p***	**HR[95% CI]**
Age (<40 vs. ≥40)	0.231		
Sex (Male vs. female)	0.156		
Smoker	<0.001	<0.001	1.336 [1.075–1.748]
Drinker	0.374		
**Primary site**			
Tongue + The mouth floor vs. others	<0.001	<0.001	2.132 [1.426–3.164]
**Pathologic tumor stage**
T3+T4 vs. T1+T2	<0.001	<0.001	3.128 [1.476–5.129]
**Tumor differentiation**
Moderate + poor vs. well	<0.001	<0.001	2.006 [1.387–3.814]
Perineural invasion	<0.001	<0.001	2.061 [1.337–3.994]
Lymphovascular invasion	<0.001	<0.001	2.116 [1.452–3.860]
Neck lymph node stage	<0.001		
N0			
N1		<0.001	1.456 [1.027–1.999]
N2		<0.001	2.375 [1.564–3.555]
N3		<0.001	3.467 [2.622–5.932]
Number of lymph node examined			
<22 vs. ≥22	0.513		
HPV positivity	0.673		
p16 positivity	0.024	<0.001	1.321 [1.048–1.733]
Positive margin	<0.001	<0.001	3.776 [1.671–5.997]
Number of positive lymph nodes	<0.001		
0			
1-2		<0.001	1.862 [1.122–2.442]
3-4		<0.001	3.189 [2.611–4.554]
≥5		<0.001	6.316 [4.673–10.227]

**Figure 3:**
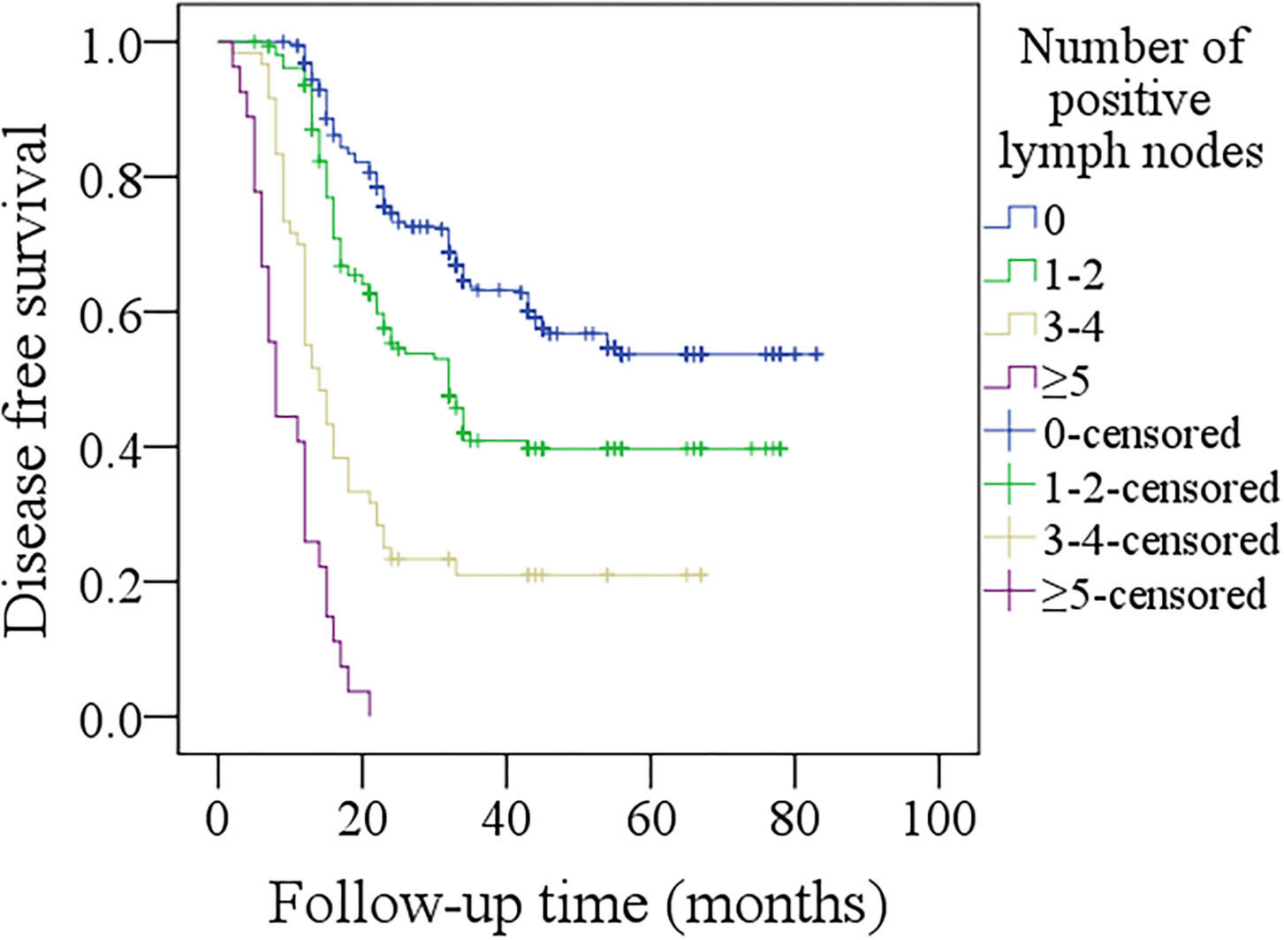
Comparison of disease-free survival among p16 negative patients with different numbers of positive lymph nodes (*p* < 0.001).

**Figure 4:**
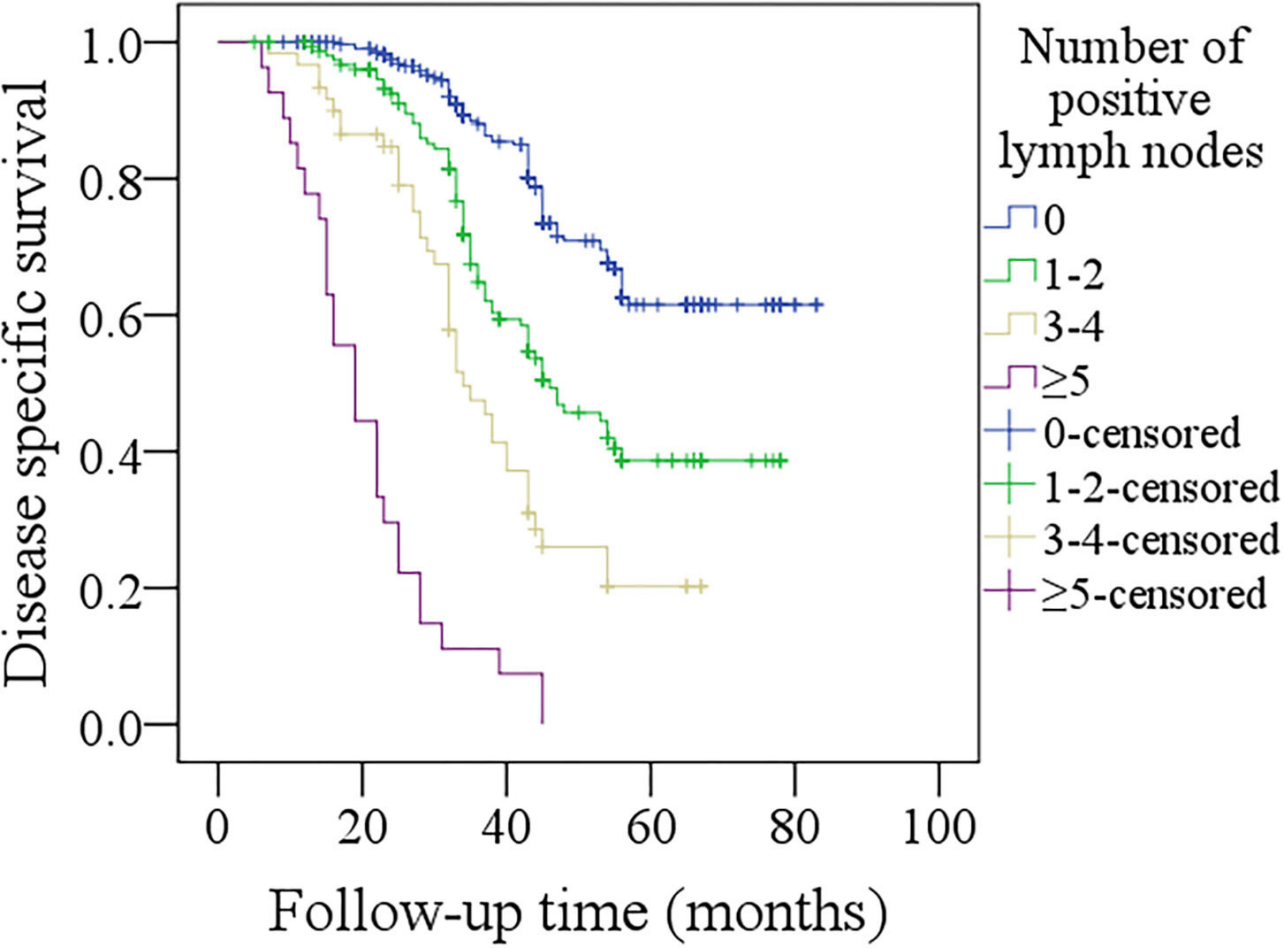
Comparison of disease-specific survival among p16 negative patients with different numbers of positive lymph nodes (*p* < 0.001).

In further sub-group analysis of patients with p16 negativity, in patients with no positive lymph nodes, the 5-year DFS rate was 52%, in patients with 1–2 positive lymph nodes, the 5-year DFS rate was 39%, in patients with 3–4 positive lymph nodes, the 5-year DFS rate was 21%, in patients with ≥5 positive lymph nodes, all patients developed recurrence within 2 years after operation. The difference was significant (Figure 3, *p* < 0.001). In patients with no positive lymph nodes, the 5-year DSS rate was 60%, in patients with 1–2 positive lymph nodes, the 5-year DSS rate was 38%, in patients with 3–4 positive lymph nodes, the 5-year DSS rate was 18%, in patients with ≥5 positive lymph nodes, all patients died within 4 years after operation. The difference was significant (Figure 4, *p* < 0.001).

**Figure 5:**
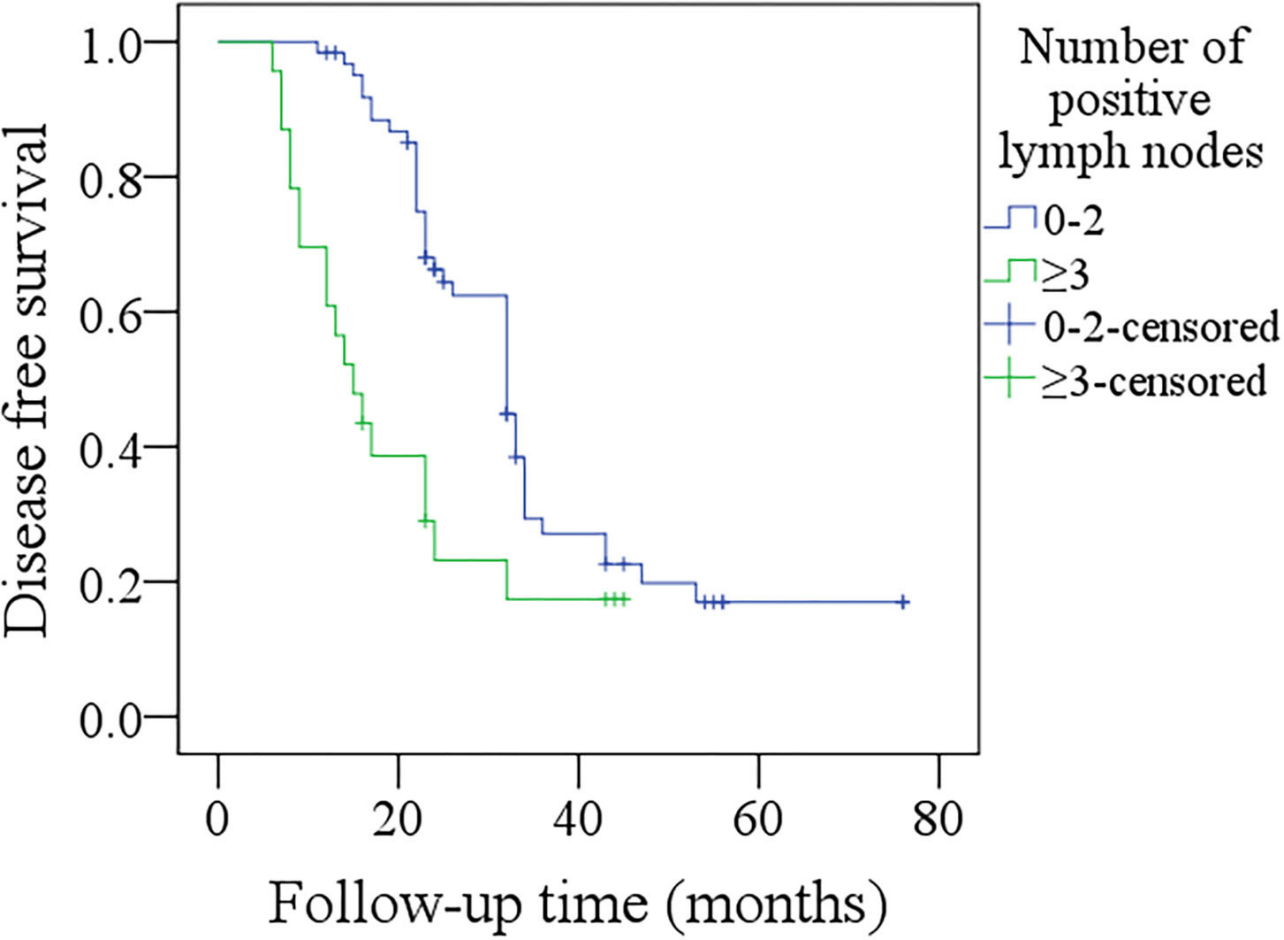
Comparison of disease-free survival among p16 positivity patients with different numbers of positive lymph nodes (*p* < 0.001).

**Figure 6:**
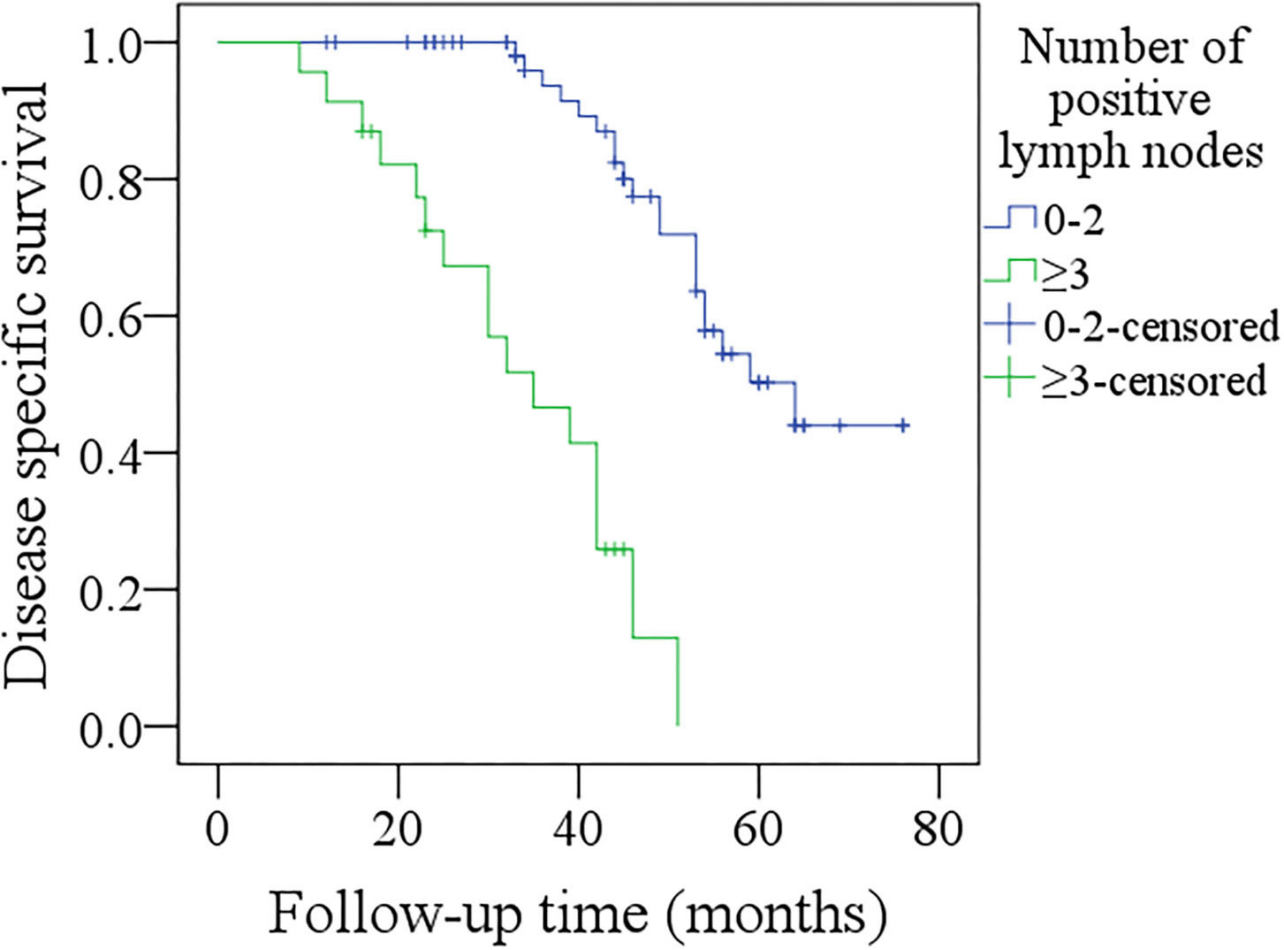
Comparison of disease-free survival among p16 positivity patients with different numbers of positive lymph nodes (*p* < 0.001).

In further sub-group analysis of patients with p16 positivity, its sample size was relatively small, therefore, we divided them into two groups based on the number of positive lymph nodes (0–2 vs. ≥3). In patients with 0–2 positive lymph nodes, the 3-year DFS rate was 41%, in patients with ≥3 positive lymph nodes, the 3-year DFS rate was 17%, the difference was significant (Figure 5, *p* < 0.001). In patients with 0–2 positive lymph nodes, the 3-year DSS rate was 84%, in patients with ≥3 positive lymph nodes, the 3-year DSS rate was 46%, the difference was significant (Figure 6, *p* < 0.001).

## Discussion

The most important finding in the current study was that we confirmed the prognostic significance of the number of positive lymph nodes in oral SCC, and the effect was unaffected by p16 status. Additionally, the number of positive lymph nodes system was superior to the 8th AJCC neck lymph node classification. It provided a reliable method to instruct adjuvant treatment and a better tool for doctor-to-patient communication.

Cervical node status was the most important prognostic factor in oral SCC, the newest version of AJCC classification was made based on analyzing the pooled database from two famous medical centers (6, 19), taking the size, number, ECS, and laterality of positive lymph nodes into consideration, although there was significant improvement in neck staging (20), apparent deficiency could not be neglected. It was previously believed that contralateral or bilateral cervical disease was associated with aggressive biologic behavior, but current evidence showed the uncommon performance tended to be contributed by unpredictable lymphatic drainage patterns rather than aggressive biology (8, 11, 21, 22).

Then some researchers introduced a revision version of neck lymph node status based on positive lymph node number. Roberts et al. (9) divided 12,437 patients with head and neck SCC into 4 groups based on the number of positive lymph nodes (0 vs. 1 vs. 2–5 vs. >5), and found patients with >5 positive lymph nodes had the worst prognosis, and the association remained independent in multivariate analysis with a lower Akaike information criterion than that in AJCC N stage. Ho et al. (23) identified 8,351 laryngohypopharyngeal patients from the National Cancer Database, in whom 56.4% had neck metastatic disease, in the survival analysis, the authors reported as number of positive lymph nodes increased, mortality risk escalated continuously without plateau, and the hazard per node was the most pronounced up to 5 metastatic lymph nodes, moreover, when accounting for positive lymph node number, the factors of the size of positive lymph nodes and contralaterality in standard nodal system had no prognostic value. The same research team selected 14,554 oral SCC patients from the National Cancer Database, and found in univariate analysis the 5-year overall survival rates were 65.3, 49.9, 41.1, 29.7, 27.5, 18.5, and 9.7% for those with 0, 1, 2, 3, 4 to 6, 7 to 9, and 10 or more positive lymph nodes, respectively, and there was still a strong relationship between the number of positive lymph nodes and overall survival after adjusting for important confounding factors (11). However, all those authors did not evaluate the effect of positive lymph node number on the DSS which was not affected by general body status. Additionally, racial difference played a significant role on cancer survival. Our study was the first to confirm the prognostic significance of positive lymph node number in DSS in oral SCC patients in China, and the model showed superiority to the AJCC N stage with higher Harrell's C-concordance index. A similar finding was also reported by Rajappa et al. (10) and Ebrahimi et al. (8).

p16 was usually used as a surrogate marker of HPV infection owing to the significant association between them in oropharynx SCC, but it was not like that in oral SCC. Harris et al. (24) noted 44% of the 25 tongue SCC patients showed p16 positivity, but none had HPV16 positivity by PCR analysis. Similarly, Poling et al. (25) found HPV positivity was only detected in 1 of the 9 cases with p16 positivity from 78 tongue SCC patients. Our finding would be consistent with these reports. The prognostic role of p16 in oral SCC was not frequently analyzed, and the existing literature showed conflicted effect results. Almangush et al. (26) previously performed a meta-analysis consisting of 174 studies and found there was no sufficient evidence to support the prognostic role of p16 in tongue SCC. Lai et al. (27) enrolled 143 patients with oral or oropharynx SCC, and determined the functional HPV presence by analyzing HPV *in situ* hybridization and p16 immunohistochemistry, in the survival analysis, the authors reported there was no significant difference of overall survival and DFS between patients with or without p16 positivity. A similar finding was also reported by Fakhry et al. (28). But Chung et al. (29) noted 62 (19.3%) of the 322 non-oropharynx head and neck SCC showed p16 positivity, and p16 over expression carried a protective effect on progression-free survival and overall survival. On the contrary, Larque et al. (30) and Dediol et al. (16) concluded p16 expression was related to worse survival in oral SCC. Our finding would also support this viewpoint. A possible explanation might be that p16 positivity meant higher number of positive lymph nodes induced by aggressive tumor behavior. More importantly, we were the first to evaluate the interaction effect of the number of positive lymph nodes and p16 positivity and note that the prognostic significance of the positive lymph node number did not alter with p16 status. The finding was novel, and provided the first possibility and feasibility of the revision nodal staging system based on the number of positive lymph nodes without considering p16 status. In a previous study by Divi et al. (31), the authors also reported the prognostic effect of the number of lymph nodes examined was not associated with p16 positivity.

Another attractive variable was the lymph node yield (LNY), which was the number of lymph nodes retrieved after neck dissection. Lemieux et al. (32) selected 4,341 patients with pN0 oral SCC, and found the mean LNY increased with tumor stage from T1 to T3, the cut-off of 22 nodes removed indicated a significant predictor of overall survival, and each additional lymph node excised was related to improved survival, and the effect maintained until 43 nodes removed. Pou et al. (33) reported that in 118 patients with cN0 head and neck SCC, metastatic disease was present in 23.73% of cases. Positive lymph node was the most likely to be detected in patients with LNY >35, and the rate was comparable in patients with LNY 26 to 35. And in patients with <18 lymph nodes, the detected rate was the lowest, then the authors concluded that the minimum for LNY was 18 for an adequate level I–III neck dissection. Kuo et al. (34) used the SEER database and found there was significant survival benefit related to ≥16 lymph nodes removed compared with lower LNY in 3097 cN0 patients, and there was survival benefit related to ≥26 lymph nodes removed compared with lower LNY in 1,268 cN+ patients. Similar findings were also reported by Divi et al. (35) and El Asmar et al. (36), but we failed to note the prognostic significance of LNY if cN0 and cN+ patients were analyzed together. There were some aspects must be considered when comprehending this finding: LNY was mainly based on the surgeon's ability of dissecting lymph nodes, the pathologist's ability of identifying the lymph nodes, and the level dissected. Treatment in academic medical center was also responsible for LNY (36). The relationship between survival and LNY was an association but not a causality, and this effect was easily affected by the neck status.

The concept of lymph node ratio (LNR), which was defined as the ratio of the number of positive lymph nodes to the number of lymph nodes examined, became more and more attentive. Hua et al. (17) enrolled 81 hypopharyngeal SCC patients, and divided these patients into three groups based on the metastatic nodes ratio (0 vs. <10% vs. >10%), and found patients with high LNR had worse prognosis in both univariate and multivariate analyses. Similar findings were also reported by Huang et al. (37) and Ding et al. (38). However, LNR was very vulnerable because of variable LNY. LNY was significantly different and increased with the number of neck levels dissected, and even in the same type neck dissection, LNY might not be the same (39), then this would lead patients with the same number of positive lymph nodes but different LNY to different neck stage. The inferiority of LNR had been verified by Ho et al. (23) and Roberts et al. (9).

Limitations in the current study must be acknowledged: firstly, the retrospective design had inherent bias; secondly, the sample size and follow-up time of patients with p16 positivity was limited, higher quality studies are needed to clarify these questions.

In conclusion, the number of positive lymph nodes are significantly associated with survival in oral SCC, and it shows superiority to AJCC N stage in predicting the prognosis. Its survival effect is not affected by p16 status.

## Data Availability Statement

The original contributions presented in the study are included in the article/supplementary material, further inquiries can be directed to the corresponding author/s.

## Ethics Statement

Henan Cancer Hospital institutional research committee approved our study, and all participants signed an informed consent agreement.

## Author Contributions

All the authors made the contribution in study design, manuscript writing, studies selecting, data analysis, study quality evaluating, manuscript revising, and read and approved the final manuscript.

## Conflict of Interest

The authors declare that the research was conducted in the absence of any commercial or financial relationships that could be construed as a potential conflict of interest.
